# Evaluation of hepatobiliary ultrasound scores in healthy dogs and dogs with liver diseases

**DOI:** 10.14202/vetworld.2019.1266-1272

**Published:** 2019-08-17

**Authors:** Sathidpak Nantasanti Assawarachan, Piyathip Chuchalermporn, Phudit Maneesaay, Naris Thengchaisri

**Affiliations:** 1 Department of Companion Animal Clinical Sciences, Faculty of Veterinary Medicine, Kasetsart University, 50 Pahonyothin Rd., Lat Yao, Chatuchak, Bangkok 10900, Thailand; 2 Endocrinology and Gastroenterology Unit, Kasetsart University Veterinary Teaching Hospital, 50 Pahonyothin Rd., Lat Yao, Chatuchak, Bangkok 10900, Thailand; 3 Radiology Unit, Kasetsart University Veterinary Teaching Hospital, 50 Pahonyothin Rd., Lat Yao, Chatuchak, Bangkok 10900, Thailand; 4 Department of Pathology, Faculty of Veterinary Medicine, Kasetsart University, 50 Pahonyothin Rd., Lat Yao, Chatuchak, Bangkok 10900, Thailand

**Keywords:** diagnosis, dogs, hepatic disease, ultrasound score

## Abstract

**Background and Aim:**

Ultrasonography is the first-line method for examining the canine liver. Hepatic ultrasound scoring systems are widely described in human medicine, yet there is no information on the use of semi-quantitative ultrasound scoring systems in canine liver diseases. This study aimed to evaluate the hepatobiliary ultrasound scores between physically healthy dogs and dogs with primary liver diseases confirmed by clinical, biochemical, and histological parameters. We also evaluated the putative correlations between ultrasound scores and ALT or ALP levels. Moreover, the severity of ultrasound scoring and fold changes in liver enzymes was also evaluated.

**Materials and Methods:**

A cross-sectional study design was conducted to compare the results of the six different parameters (liver surface, echogenicity of parenchyma, nodularity of parenchyma, gallbladder wall thickness, amount of gall sludge, and visibility of bile duct) of ultrasound scores between dogs with and without liver disease.

**Results:**

Our results showed that 17.4%, 88.2%, and 100% of dogs with liver diseases were identified according to the ultrasound severity classified as mild (total score 0-2), moderate (total score 3-5), and severe (total score 6-12). Approximately 30% of patients with chronic hepatitis, the most common canine liver disease, presented with normal or mild ultrasound score category, whereas most of the patients with vacuolar hepatopathy and steroid-induced hepatopathy due to secondary reactive changes had moderate-to-severe ultrasound score category. There were 75% of patients with tumor and 80% of patients with hepatic fibrosis that were identified with severe ultrasound score category. Dogs with moderate-to-severe ultrasound scores had significant liver enzyme elevation (both alanine aminotransferase [ALT] and alkaline phosphatase [ALP]) compared to those of dogs with mild ultrasound scores. Ultrasound score was moderately associated with ALT and highly associated with ALP levels (p=0.553 and p=0.730, respectively).

**Conclusion:**

Our semi-quantitative, simplified ultrasonographic scoring system may have potential to be used as a screening tool to detect some groups of liver diseases.

## Introduction

Hepatobiliary diseases are among the leading causes of morbidity and mortality in small animals. Identifications of elevated liver enzymes are often the first parameters indicating the presence of liver diseases in dogs [1-3]. However, non-hepatic disorders involving endotoxins or infectious agents may affect liver enzyme activity [2,4]. Thus, medical history, signalment, physical findings, laboratory testing, imaging techniques, and histopathology are all needed to diagnose the diseases [5]. Before a biopsy is performed for definite diagnosis of canine liver diseases, several non-invasive strategies are often accomplished as potential diagnostic alternatives. A marker of hepatocyte injury that is more sensitive than alanine aminotransferase (ALT) is also needed to detect liver diseases early. The studies revealed that serum microRNA-122 was more sensitive than ALT in identifying copper-induced hepatocellular injury [6], and acute and chronic hepatitis [7]; however, its use in clinical settings needs further study. In addition, hyaluronic acid was found to have good sensitivity for the diagnosis of canine cirrhotic liver disease [8]. Ultrasonography is the first-line and widely available method for examining the canine liver [9,10]. The ultrasonographically guided collection of samples for cytology or histology is an important part of an in-depth evaluation of hepatobiliary diseases [1]. Unfortunately, a definitive diagnosis of canine hepatic disease, especially infiltrative diseases, cannot be made from ultrasonographic appearance alone. The previous studies have found that no relationship exists between any of the ultrasonographic criteria and microscopic diagnoses or laboratory values [11-13]. Sensitivity of ultrasonography for hepatic disease is generally questionable due to the high variation among types of lesions. Studies have found that ultrasonography had a sensitivity of 0% for diagnosing metastatic mast cell tumors [14], 44% for round cell neoplasia, 48% for hepatitis, 67% for vacuolar hepatopathy [13], 73% for metastatic lymphoma [15], 80% for hepatocellular carcinoma, 84% for cholangitis/cholangiohepatitis [16], and 86% for steroid hepatopathy [13].

All the ultrasonographic features of the previous studies in canine patients were considered based on the presence of abnormal liver appearance. Ultrasound scoring systems have been widely used in human medicine to diagnose various liver diseases and provide objective assessments to evaluate the severity of the tissue pathology at clinical setting [11,13,17,18]. To the authors’ knowledge, no studies have evaluated the use of scoring systems in small animal clinical settings.

This study aimed to evaluate the hepatobiliary ultrasound scores between physically healthy dogs and dogs with primary liver diseases confirmed by clinical, biochemical, and histological parameters. We also evaluated the putative correlations between ultrasound scores and ALT or ALP levels. Moreover, the severity of ultrasound scoring and fold changes in liver enzymes was also evaluated.

## Materials and Methods

### EthiScal approval

The use of animals in this study was approved by the Kasetsart University Institutional Animal Care and Use Committee (ACKU61-VET-012), Thailand. Animals with elevated liver enzymes due to non-primary liver diseases were excluded from the study.

### Study animals

A total of 80 dogs, 40 physically healthy dogs and 40 dogs with liver diseases, were evaluated with each owner’s informed consent. We evaluated 80 dogs that were patients of Kasetsart University Veterinary Teaching Hospital over 2 years (January 2016-January 2018) and informed consent was obtained from all owners. Of these 80 dogs, 40 were physically healthy and 40 were determined to have liver diseases. The equal weight of sample sizes in both negative and positive group helps to increase the precision and accuracy of the ultrasound comparison.

### Sampling

The sample for the liver biopsy was obtained from the diseased group during a routine diagnostic procedure using 14G spring-loaded needles (Argon, Frisco, TX, USA) under ultrasound guidance on the same day as ultrasonographic scanning. A Thai board-certified pathologist who was unaware of the ultrasound findings performed histology of the livers to confirm the specific liver diseases. The blood were collected through cephalic or saphenous vein from both the control and diseased dogs for blood urea nitrogen (BUN), creatinine, ALT, alkaline phosphatase (ALP), total protein, and albumin.

### Ultrasonography

Ultrasonography of all dogs was performed using a real-time scanner (GE, Fairfield, CT, USA) with a 13 MHz broadband, linear transducer. An experienced veterinary radiologist performed all examinations in a blinded fashion. All dogs fasted at least 12 h before the procedure. Dogs were scanned in lateral or sternal recumbency and were manually restrained (control group) or underwent general anesthesia (disease group) before an ultrasound-guided needle biopsy. During the ultrasound, all lobes of the liver were evaluated. The ultrasound parameters and their assigned scoring system, which were a modified version adopted from previously published literature, are depicted in Table-1 [11,17-19]. Ultrasonographic features were categorized on the basis of (1) liver surface, (2) parenchymal score (echogenicity of parenchyma and nodularity of parenchyma), and (3) biliary score (gallbladder wall thickness, amount of gall sludge, and visibility of bile duct). Each parameter was scored with a 0, 1, 2, or 3, and the ultrasound score from each group was calculated as the sum of the scores of these six parameters.

**Table 1:** Hepatobiliary ultrasonographic scores for evaluation of canine hepatobiliary systems with a total score ranging from 0 to 12.

Score type	Clinical features	Scores			

		0	1	2	3
Surface score	Liver edge/border (0-2)	Sharp	Mildly blunt	Blunt	
Parenchymal score	Parenchymal echogenicity (0-3)	Normal	Hypo-/hyper-echogenicity	Inhomogeneous (mildly coarse)	Heterogeneous (coarse)
	Nodularity of parenchyma (0-2)	Smooth	Mildly irregular	Irregular	
Biliary score	Gallbladder wall thickness (0-1)	<2 mm	>2 mm		
	Gall sludge (0-3)	Normal	Increase	Stellate sludge	Stone
	Bile duct visibility (0-1)	No	Yes		

### Statistical analysis

The data analysis was performed using GraphPad Prism Version 6 (GraphPad Software Inc., La Jolla, CA, USA). All parameters including dog characteristics, biochemical values, and ultrasound scores were compared between healthy dogs and dog with liver diseases using Student’s t-test. Data were expressed as mean ± standard deviation. The statistically significant level was set at p<0.05. Spearman’s rank-sum correlation test (p) was applied to determine the relationship between ultrasound scores and liver enzymes (ALT and ALP).

## Results

A total of 40 physically healthy dogs and 40 dogs with liver diseases were evaluated. Each group had 20 male and 20 female dogs. The average ages were 4.9 and 10 years old, respectively, in the healthy group and the diseased group. The dog’s characteristics such as sex, age, and size are summarized in Table-2. Biochemical evaluations including BUN, creatinine, ALT, ALP, total protein, and albumin are summarized in Table-3. Diseased dogs had significantly higher ALT and ALP than the dogs in the healthy group (p<0.05 and p<0.01, respectively) (Table-3). Of the 40 dogs with liver disease, there were 23 (57.5%) with chronic hepatitis, 6 (15.0%) with vacuolar hepatopathy, 5 (12.5%) with hepatic fibrosis, 2 (5.0%) with steroid-induced hepatopathy, 1 (2.5%) with hemangiosarcoma, 1 (2.5%) with hepatoma, 1 (2.5%) with hepatocellular carcinoma, and 1 (2.5%) with sinusoidal ectasia. A moderate relationship between the ultrasound scores and the level of ALT was found (p=0.553, p<0.01) (Table-4). In addition, a high correlation between ultrasound scores and ALP levels was found (p=0.730, p<0.01) (Table-4). A mild negative correlation between ultrasound scores and creatinine levels was also identified (p=−0.399, p<0.01) (Table-4).

**Table 2:** General characteristics of the 40 healthy dogs and the 40 hepatic disease dogs.

Parameter	Healthy	Hepatic disease
N	40	40
Sex		
Male	20	20
Female	20	20
Average age, years (mean±SD)	4.9±2.8	10.0±3.3
Body size		
Small	14	22
Medium	2	11
Large	24	7

SD=Standard deviation

**Table 3:** Average serum chemistry values in the dogs.

Parameter	Healthy	Hepatic disease	Normal value	Unit
BUN	17.3±7.2	18.8±9.3	10-26	mg%
Creatinine	1.0±0.2	0.9±0.3	0.5-1.3	mg%
ALT	35±9	361±783*	6-70	IU/L
ALP	51±51	1052±1945**	8-76	IU/L
Total protein	6.7±0.6	7.1±0.9*	5.3-7.8	mg%
Albumin	3.2±0.3	3.3±0.5	2.3-3.2	mg%

ALP=Alkaline phosphatase, ALT=Alanine aminotransferase, BUN=Blood urea nitrogen.

* p<0.05 compared to Healthy,

** p<0.01 compared to Healthy

**Table 4:** Spearman’s rank correlation coefficient (p) between ultrasound scores and serum chemistry values.

Parameters	ALT	ALP	BUN	Creatinine
ALT	-	0.543** (0.387, 0.699)	0.109 (−0.125, 0.344)	−0.192 (−0.408, 0.022)
Ultrasound score	0.553** (0.410, 0.697)	0.730** (0.623, 0.836)	0.181 (−0.041, 0.403)	−0.399** (−0.604, −0.193)

** p<0.001. ALP=Alkaline phosphatase, ALT=Alanine aminotransferase, BUN=Blood urea nitrogen

**Figure-1:**
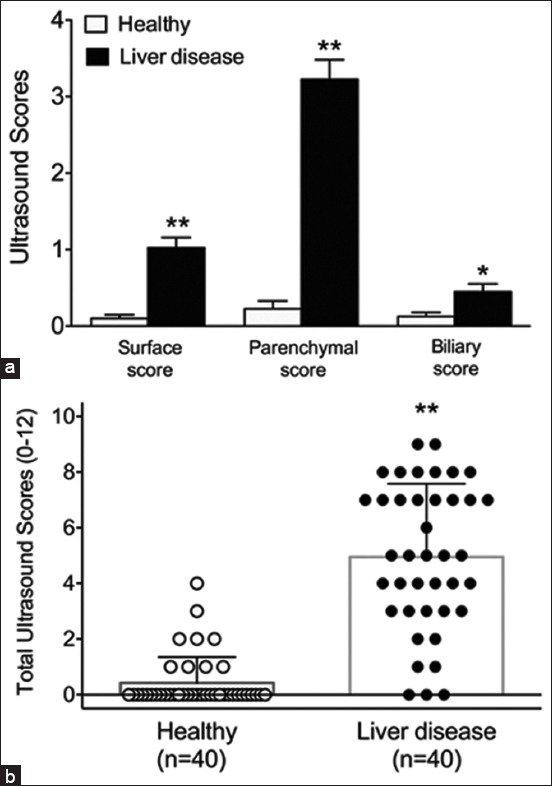
Ultrasound scores of the healthy and diseased group. (a) Histogram illustrating the average scores of each ultrasound criterion of the healthy and diseased group. (b) Scatter plot illustrating the total ultrasound criterion of the healthy and diseased group. *p<0.05 compared to healthy group; **p<0.01 compared to healthy group.

Dogs with liver disease had significantly higher ultrasound scores than the healthy dogs, including liver surface score (1.03±0.83 vs. 0.10±0.30, p<0.01), parenchymal score (3.23±1.62 vs. 0.23±0.66, p<0.01), and biliary score (0.45±0.64 vs. 0.13±0.33, p<0.05) (Figure-1a). Moreover, the average total ultrasound score (0–12) of dogs with liver disease (4.70±2.52) was higher than that of the control dogs (0.45±0.96, p<0.01) (Figure-1b).

**Figure-2:**
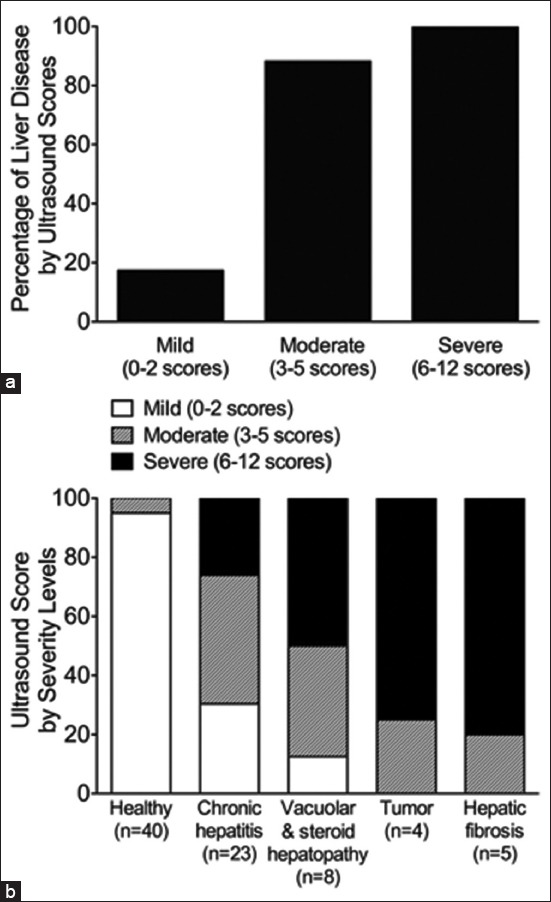
Three severity levels of ultrasound score for diagnosis of liver diseases. (a) Histogram reveals the percentage of animals with liver disease detection at different ultrasound severity levels. (b) Histogram demonstrates the three severity levels of the ultrasound score in healthy group and four subgroups of liver diseases.

All animals were then divided into three different severity categories of total ultrasound score, namely, mild (score 0-2), moderate (score 3-5), and severe (score 6-12). The percentage of diseased dogs versus all animals in each category is shown in Figure-2a. There were 17.4%, 88.2%, and 100% of dogs with liver diseases identified by mild, moderate, and severe ultrasound severity categories, respectively. More than 95% of healthy dogs had mild ultrasound score (Figure-2b). Approximately 30% and 40% of patients with chronic hepatitis presented with mild and moderate changes in liver ultrasonographic features, respectively. Interestingly, about 50% of animals with vacuolar hepatopathy and steroid-induced hepatopathy can be classified as severe ultrasound score category. All animals with tumor and hepatic fibrosis had moderate-to-severe ultrasound score category, with approximately 75-80% of patients classified as severe score, indicating that the diseases significantly affected liver parenchymal structure (Figure-2b).

**Figure-3:**
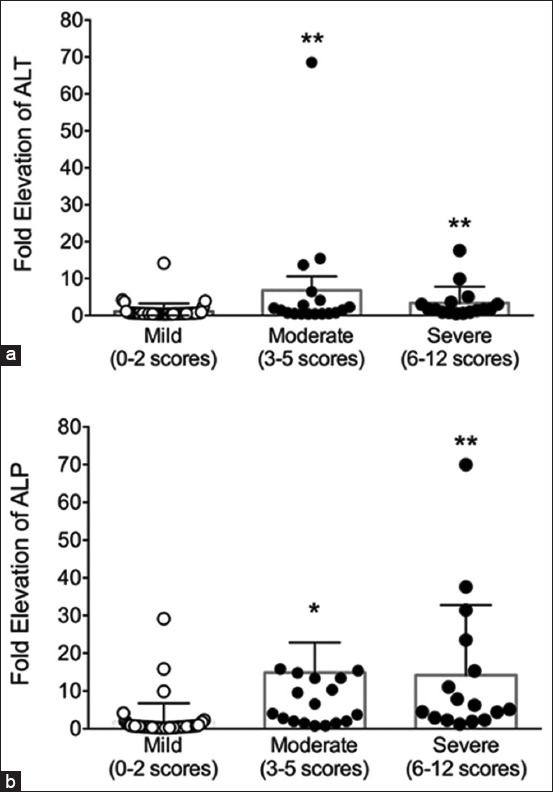
Diagram illustrating fold increase in alanine aminotransferase (ALT) or alkaline phosphatase (ALP) according to the ultrasound score. (a) Scatter plot demonstrates fold elevation of ALT according to the three severity levels of ultrasound scores. (b) Scatter plot demonstrates fold elevation of ALP according to the three severity levels of ultrasound scores. *p<0.05 compared to group with mild ultrasound score; **p<0.01 compared to group with mild ultrasound score.

Diseased animals with moderate and severe ultrasound score categories had significantly higher serum ALT concentration (p<0.01) than those with mild ultrasound score category (Figure-3a). In addition, patients with moderate and severe ultrasound score categories had significantly higher serum ALP concentration (p<0.05 and p<0.01, respectively) than those with mild ultrasound score category (Figure-3b).

## Discussion

Several investigators proved that routine clinical ultrasound does not have sufficient accuracy for detecting specific canine liver diseases [11-13]; ultrasonography remains the most common tool to evaluate a patient’s liver. No previous studies have assessed the semi-quantitative ultrasonographic scoring systems in canine liver diseases. In the present study, we modified previously published ultrasound scoring systems in human patients [11,13,17,18], consisting of liver edge, parenchymal echogenicity, nodularity of parenchyma, and gallbladder and bile duct appearance, to assess if the system may aid diagnosis of canine hepatobiliary diseases.

In this study, the dogs with liver disease were older than the dogs in the healthy group. The different age distribution between two groups may be attributed to the fact that liver diseases included chronic hepatitis and hepatocellular carcinoma commonly are found in older dogs [3,20-24]. Chronic hepatitis was the most common liver diseases found in this study (57.5%). American cocker spaniel, Cairn terrier, Dalmatian, Doberman pinscher, English cocker spaniel, English springer spaniel, Great Dane, Labrador retriever, and Samoyed have an increased risk for developing chronic hepatitis [20]. Therefore, age and breed difference between two groups could affect the ultrasound scoring. The sex distribution was equal between groups. The diseased dogs showed significantly higher liver enzymes ALT and ALP than the healthy group.

We demonstrated that the diseased group accounted for about 17% of all animals with mild ultrasound score category, whereas they accounted for 80-100% of animals having moderate and severe categories. Animals with a significant abnormality of hepatobiliary ultrasonography (moderate-to-severe score) most likely have liver lesions. However, chronic hepatitis was presented with up to 70% of mild-to-moderate ultrasound score categories. These findings indicated that chronic hepatitis, the most common liver diseases in dogs [13] can be presented with normal to mild changes of liver ultrasonographic features at clinical setting. Caution must be taken when considering liver biopsy, especially in patients with chronically elevated liver enzymes with normal to mild ultrasound score category to avoid a misdiagnosis of chronic hepatitis.

Although animals with elevated liver enzymes due to extrahepatic causes were excluded in our study, 15% of all patients were histopathologically diagnosed as vacuolar hepatopathy. These findings are likely due to the secondary reactive change from glucocorticoid excess or stress-induced hypercortisolemia as a result of underlying primary liver disease [24]. Other studies demonstrated 10-19% of patients with vacuolar hepatopathy from all hepatic diagnoses [13,24,25]. In this study, there were 90% of patients with vacuolar hepatopathy or steroid-induced hepatopathy animals classified in moderate-to-severe ultrasound score categories, with at least a half of the patients presented with severe ultrasound score. Therefore, abnormal ultrasound score might not always indicate primary liver diseases and the need for specific treatment. Non-primary liver diseases such as vacuolar hepatopathy may be found in individuals with chronically elevated liver enzyme activity and significant liver ultrasonographic changes. However, it should not be ignored because a fulminant hepatic dysfunction can be developed secondary to severe vacuolar hepatopathy [24]. In addition, about 75-80% of patients with liver fibrosis or tumor had total ultrasound score in severe category. It may be implied that hepatic fibrosis and tumor are unlikely to be identified in patients with mild ultrasound score category. Nonetheless, the actual values as well as specific cutoff point of ultrasound scores for prediction of the liver disease remain unknown. The role of ultrasound score for prognosis of canine liver disease remains elusive and needs further evaluation.

ALT and ALP are among the most common liver enzymes abnormalities in patients with liver disease. Increased ALT and ALP serum activity indicates damage of the hepatocellular membrane and cholestasis, respectively [2]. In this study, we demonstrated that dogs with moderate-severe ultrasonographic scores had a significant elevation of liver enzymes, ALT and ALP, compared to those of dogs with mild ultrasonographic scores. The relationship between ultrasound scores and ALT or ALP levels was positively correlated. Degree of hepatocellular damage and cholestasis indicated by elevation of ALT or ALP levels may lead to ultrasonographic changes in the liver. Our data may support the usefulness of ultrasound scoring system for the determination of the severity of liver damage and cholestasis. However, it has been shown that the magnitude of liver enzymes abnormality does not always correlate well with the severity of hepatic diseases [26].

The limitations of this study include that it evaluated patients with mixed liver disease etiologies and histological characteristics. The categorical scores used in the present study provide the overall views rather than a specific type for certain liver disease. In addition, choosing the criteria for the objective ultrasonographic assessment of liver diseases in dogs from the wide range of ultrasound parameters and variable recommended algorithms is challenging. The results reported in this study also may be overestimated because dogs with ultrasonographic lesions are more likely to be biopsied than those without lesions. The limitation of the present study included the small sample size in both control and liver disease group. Therefore, more studies are needed to evaluate the efficacy of the ultrasound scoring system to separate specific liver diseases. Another limitation is that the scans in this study were performed by the same sonographer and the same scanner. It is common knowledge that ultrasound interpretation varies among readers [26,27]. Additional validation studies are necessary to evaluate the intraobserver and interobserver variations, wherein readers can be trained to uniformly interpret ultrasound images using the ultrasound scoring system presented here. However, one scanner may have reduced variation from operator bias and non-uniformity in the quality of the ultrasound [26,27]. In addition, our study evaluated real-time images on high-resolution screens, which may have been an advantage compared with previous retrospective studies that relied on static images. The last limitation is that liver biopsy was not done for dogs in the healthy control group. Clinical data, biochemical data, as well as ultrasonographic features revealed no evidence of liver lesions in the control dogs. It should be noted that healthy dogs were younger than dogs with liver diseases. Young dogs have been shown to have a lower risk of developing liver disease than older dogs [20,22-24,28].

## Conclusion

Our semi-quantitative, simplified ultrasonographic scoring system assessing six different parameters may have potential to be used as a screening tool to detect some groups of liver diseases with structural changes including tumor and fibrosis, and a good diagnostic tool for evaluation of liver damage. Animals with severe ultrasound score category (total score 6-12) were also suggested for further diagnosis using liver biopsy. Beneficial role of our ultrasound scoring system to separate specific liver diseases still remained unclear. Sensitivity and specificity of ultrasound scoring system for specific liver diseases require a larger sample size. Moreover, the combination of different echographic parameters may ultimately improve the performance of the test. In conclusion, we have proved here that hepatobiliary ultrasonographic scores are useful for evaluation of canine hepatobiliary diseases.

## Authors’ Contributions

SNA designed the study, conducted the experiment, and wrote the manuscript. PC performed ultrasound and calculated ultrasound score. PM performed histological analysis. NT designed the study and performed statistical analysis. All authors contributed to drafting and revision of the manuscript. All authors read and approved the final manuscript.
